# Shear-induced enhancements of crystallization kinetics and morphological transformation for long chain branched polylactides with different branching degrees

**DOI:** 10.1038/srep26560

**Published:** 2016-06-01

**Authors:** Junyang Wang, Jing Bai, Yaqiong Zhang, Huagao Fang, Zhigang Wang

**Affiliations:** 1 CAS Key Laboratory of Soft Matter Chemistry, Department of Polymer Science and Engineering, Hefei National Laboratory for Physical Sciences at the Microscale, University of Science and Technology of China, Hefei, Anhui Province 230026, P. R. China; 2 Provincial Key Laboratory of Advanced Functional Materials and Devices, Institute of Polymer Materials and Chemical Engineering, School of Chemistry and Chemical Engineering, Hefei University of Technology, Hefei, Anhui Province 230009, P. R. China

## Abstract

The effects of long chain branching (LCB) degree on the shear-induced isothermal crystallization kinetics of a series of LCB polylactides (LCB PLAs) have been investigated by using rotational rheometer, polarized optical microscopy (POM) and scanning electron microscopy (SEM). Dynamic viscoelastic properties obtained by small-amplitude oscillatory shear (SAOS) tests indicate that LCB PLAs show more broadened relaxation time spectra with increasing LCB degree. Upon a pre-shear at the shear rate of 1 s^−1^ LCB PLAs show much faster crystallization kinetics than linear PLA and the crystallization kinetics is enhanced with increasing LCB degree. By modeling the system as a suspension the quantitative evaluation of nucleation density can be derived from rheological experiments. The nucleation density is greatly enhanced with increasing LCB degree and a saturation in shear time is observed. Crystalline morphologies for LCB PLAs observed by POM and SEM demonstrate the enhancement of nucleation density with increasing LCB degree and a transformation from spherulitic to orientated crystalline morphologies. The observation can be ascribed to longer relaxation time of the longest macromolecular chains and broadened, complex relaxation behaviors due to the introduction of LCB into PLA, which is essential in stabilizing the orientated crystal nuclei after pre-shear.

Polylactide (PLA) with excellent performance in biocompatibility, biodegradability, renewability and mechanical properties has attract great industrial interests, especially in biomedical and commodity applications^1^,^2^,^3^. Unfortunately, due to its inherent slow crystallization kinetics PLA crystallizes much slowly during the melt crystallization process, as compared to other semicrystalline polymers, such as polypropylene (PP) and polyethylene (PE)^4^. A much low crystallinity of PLA is achieved during industrial processing where a large cooling rate exists, leading to some undesirable properties such as the low barrier and thermal resistance property^5^,^6^,^7^. To improve the crystallization kinetics and the ultimate crystallinity of PLA, several methods have been adopted such as annealing above the glass transition temperature, *T*_g_ where cold crystallization occurs^8^ and/or introducing effective nucleating agents such as talc and carbon nanotubes^6^,^9^,^10^,^11^,^12^,^13^. However, these methods show limitations in industrial processing, for which high cooling rate and shear gradient coexist.

Introducing long chain branched (LCB) structure into linear PLA to produce long chain branched polylactide (LCB PLA) has been reported in recent years, originally to improve the melt strength and molecular mass favoring the foaming of PLA^14^,^15^,^16^,^17^,^18^. The quiescent isothermal crystallization behavior of LCB PLA is expected to be greatly enhanced as compared with linear PLA^15^,^16^,^17^,^19^. Wang *et al.* claimed that long chain branched PLA crystallized faster than the linear one, while the ultimate crystallinity of branched PLA was found to be reduced, which could be tailored by controlling the branching degree and the length of the branched chains^16^. Nofar *et al.* studied the quiescent crystallization behavior of linear PLA and LCB PLAs with different branching degrees and found that LCB PLAs crystallized much faster than linear PLA, for which the branched chains play as a role of nucleating sites^18^. The study on crystallization kinetics of linear PLA and bimodal LCB PLA in our group has demonstrated that the crystallization kinetics is greatly accelerated for LCB PLA as compared to linear PLA and the enhancement of crystallization kinetics is dominated by nucleation with the spherulitic growth rate of LCB PLA decreasing slightly^20^,^21^. However, a systematic study on the connection between microscopic molecular structures and the crystallization behaviors of LCB PLA is still lacking.

Besides the introduction of LCB structures, shear flow is an efficient approach to enhance crystallization of PLA, which exists inevitably in polymer processing (e.g., extrusion, injection molding, blow molding)^21^,^22^. Flow-induced crystallization of various semicrystalline polymers such as PP and PE has been evidenced, in which the high molecular mass chains have been demonstrated to be of vital importance to the formation of shish-kebab morphology^22^,^23^,^24^,^25^,^26^,^27^. Thus, analogous effect of the LCB structure on shear-induced crystallization behaviors of long chain branched polymers can be expected. Agarwal *et al.* studied the shear-induced crystallization of long chain branched isotactic polypropylene (LCB iPP) and reported that both the crystal fraction and crystallization kinetics of LCB iPP were improved compared to that of linear iPP^23^. Heeley *et al.* claimed that for the shear-induced crystallization of blends of monodisperse hydrogenated polybutadiene and a high-molecular-mass LCB polybutadiene comb-shaped additive to form shish-kebab morphology, the amount of LCB combs must be above the overlap concentration, c* to guarantee the mutual overlapping of LCB combs and the comb Weissenberg number, *W*_i_^comb^ must be in the strong stretch region^25^. For linear PLA characterized with intrinsic semirigid macromolecular backbone, a fast relaxation after shear flow occurs, which reduces the efficiency of shear flow in altering the nucleation ability and crystalline morphology of PLA. Fang *et al.* prepared bimodal LCB PLA through *γ* irradiation and found that LCB PLA showed higher nucleation density and faster crystallization kinetics as compared with linear PLA and for sufficient shear time a morphological transformation from spherulite to row-like crystals occurs^21^. In general, the branching degree of LCB polymers, which leads to distinct chain topological structures and complex chain relaxation behavior, is an essential factor affecting the crystallization behaviors of LCB polymers. However, a detailed study on the effect of branching degree on shear-induced crystallization of LCB PLA has been rarely reported, which is of great significance for meditating PLA crystallization and the corresponding properties of industrial PLA products.

In this work the shear-induced crystallization kinetics of LCB PLAs with different branching degrees was systematically investigated. The viscoelastic properties of LCB PLAs were explored by rheometry, from which the presently adopted shear conditions could be determined. Shear-induced isothermal crystallization processes of linear PLA and LCB PLAs were followed by rotational rheometer and *in-situ* polarized optical microscopy (POM) observation, respectively. The quantification of crystallization kinetics and nucleation density for LCB PLAs with different branching degrees is made by modeling the crystallizing PLA samples as a suspension-like system, and is correlated to the observed morphological characteristics. Finally, the results are explained by a simple picture, for which how the relaxation of LCB structures with different branching degrees after experiencing different shear conditions influences the subsequent crystallization kinetics, nucleation densities and crystalline morphologies is described.

## Results and Discussions

### Rheological properties of linear PLA and LCB PLAs with different branching degrees

**Figure 1:**
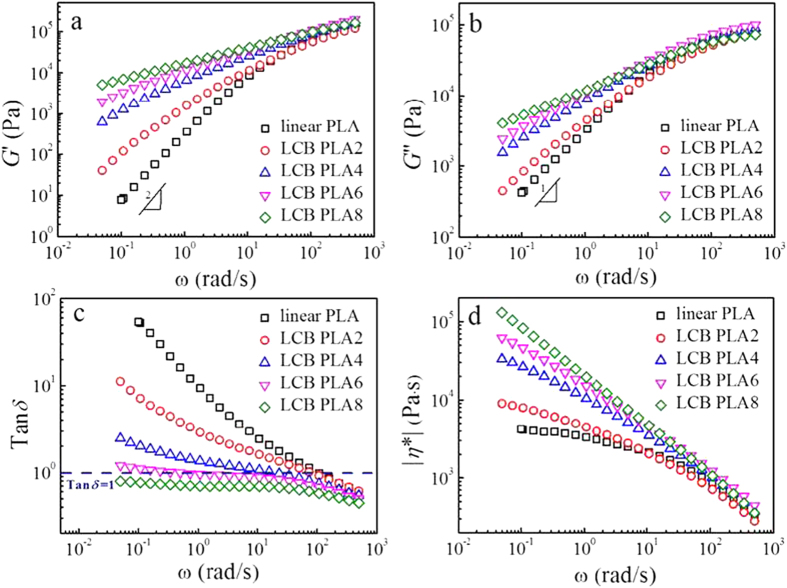
Changes of (**a**) storage modulus, *G′*, (**b**) loss modulus, *G′′*, (**c**) loss tangent, tan *δ*, and (**d**) complex viscosity, |η*|, respectively as functions of angular frequency, *ω* for linear PLA and LCB PLAs at 180 °C.

The melt rheological properties can be greatly influenced by the changes of topological molecular structures, such as the LCB structures^16^,^17^,^25^,^28^,^29^. The changes of storage modulus, *G*′, loss modulus, *G*′′, loss tangent, tan*δ* and complex viscosity, |η*| as functions of angular frequency, *ω* for linear PLA and LCB PLAs at 180 °C are shown in Fig. 1. It can be found that in the highest frequency region the *G*′ and *G*′′ of linear PLA and LCB PLAs possess the identical values, while in the low frequency region the *G*′ and *G*′′ for LCB PLAs increase considerably with increasing branching degree. In general, in the terminal region the *G*′ and *G*′′ follow the well-known frequency dependency, i.e., *G*′ ∝ *ω*^2^ and *G*′′ ∝ *ω*, for which only the longest relaxation times contribute to the viscoelastic behavior^21^,^23^. For LCB PLAs, the decreasing terminal slope, i.e., the reducing frequency-dependency of *G*′ and *G*′′ in the terminal region with increasing branching degree is apparent, indicating the more and more dominant elastic behavior. The frequency dependence of loss tangent, tan*δ* is illustrated in Fig. 1c. The reduced frequency dependence of tan*δ* with increasing branching degree in the low frequency region suggests a transition from a liquid-like behavior for linear PLA to a gel-like behavior for LCB PLAs. Eventually plateau regions are reached for LCB PLA6 and LCB PLA8 with higher branching degrees. The shear-thinning behavior can be greatly influenced for LCB PLAs as compared to linear PLA. The frequency dependence of complex viscosity, |η*| is shown in Fig. 1d. In the low frequency region, |η*| increases with increasing branching degree. However, as compared with linear PLA the absence of frequency-independent region for LCB PLAs in the experimental frequency range suggests the transition from a Newtonian plateau for linear PLA to a power-law regime for LCB PLAs. The shear-thinning behavior for LCB PLAs is greatly enhanced by increasing the branching degree. Therefore, the much more pronounced nonterminal behaviors for LCB PLAs with higher branching degrees suggest that the relaxation time can be greatly increased by improving the branching degree of LCB PLAs, which is consistent with the results for LCB PLAs prepared with other methods^16^,^18^,^30^.

**Figure 2:**
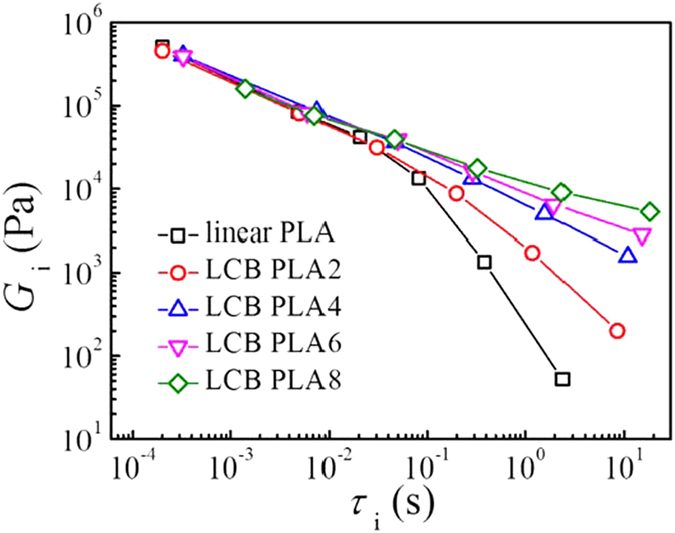
Relaxation behaviors of linear PLA and LCB PLA melts measured at 180 °C.

To evaluate the effects of introduction of LCB structures and branching degree on the relaxation behaviors for LCB PLAs, the discrete Maxwell relaxation time spectrum (*G*_i_, *τ*_i_) is adopted, which expresses *G*′ and *G*′′ as follows:


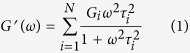



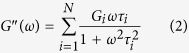


where *G*_i_ is the modulus corresponding to the relaxation time, *τ*_i_. The method in a previous work by using the Trios Software (TA Instruments) was applied to determine the parameters of the discrete relaxation spectrum by fitting equations (1) and (2) to the *G*′ and *G*′′ data in Fig. 1 21,31. The sets of *G*_i_ and *τ*_i_ for linear and LCB PLAs derived from the discrete relaxation time spectrum are presented in Fig. 2. The result shows that the shortest relaxation modes are similar for all the LCB PLAs, except for LCB PLA8 with higher shortest relaxation time. However, increasing the branching degree results in a broadening of the relaxation spectrum in the longest relaxation time region, and the longest relaxation time increases from 2.39 s for linear PLA to 18.46 s for LCB PLA8.

Before an application of any shear flow to LCB PLA melts for studying the shear-induced crystallization, the appropriate shear conditions must be determined. The transitions between different shear regimes for shear-induced crystallization are usually defined by two characteristic Weissenberg numbers, *Wi*_rep_ = 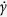
*τ*_rep_ and *Wi*_s_ = 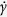
*τ*_s_, on the basis of the reptation time, *τ*_rep_ and the Rouse time or stretch relaxation time, *τ*_s_, respectively^24^,^26^,^27^,^32^. To guarantee the formation of shish-kebab morphology, the shear rate, 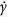 must lie in the strong stretch regime, i.e., *Wi*_rep_ > 1, *Wi*_s_ > 1. There are strong experimental evidences that the high molecular weight (HMW) tail of the molecular mass distribution (MMD) dominates the shear-induced crystallization dynamics^33^,^34^,^35^,^36^. The critical shear rates for the different shear regimes of linear PLA can be determined from *τ*_rep_^HMW^, the longest reptation time of the relaxation time spectrum shown in Fig. 2. The detailed calculation procedure can be found in the Supplementary Information. The critical shear rate for onset of the molecular chain stretching of linear PLA at 130 °C is 1.1 s^−1^. As for LCB PLAs, the much higher longest relaxation times can lead to dramatic decrease in the critical shear rate values. Therefore, the molecular stretching regime could be easily reached for LCB PLAs when the shear rate of 1 s^−1^ was applied. Furthermore, to fulfill the strong molecular stretching, the stretch ratio, *λ*, must exceed the critical temperature-dependent value, λ*(*T*)^37^,^38^,^39^. As a consequence, the shear conditions with different shear times at the shear rate of 1 s^−1^ were chosen to examine the effects of shear flow on the crystallization behaviors for linear PLA and LCB PLAs with different branching degrees.

### Shear-induced isothermal crystallization kinetics for linear PLA and LCB PLAs

**Figure 3:**
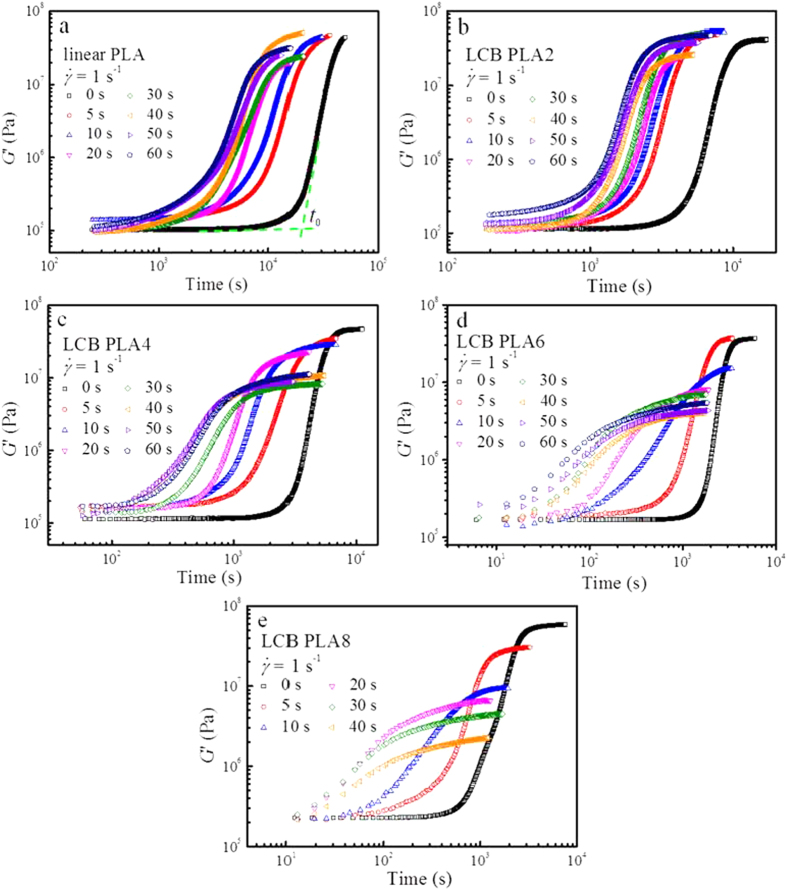
Evolutions of storage modulus, *G*′ during isothermal crystallization at 130 °C for (**a**) linear PLA, (**b**) LCB PLA2, (**c**) LCB PLA4, (**d**) LCB PLA6, and (**e**) LCB PLA8 under the quiescent and various shear conditions.

Rheology can be well applied to follow the crystallization kinetics of semicrystalline polymers, which shows several advantages as compared to the conventional methods such as differential scanning calorimetry (DSC) and polarized optical microscopy (POM)^37^,^40^,^41^,^42^,^43^. The evolutions of storage modulus, *G*′ during isothermal crystallization for linear PLA and LCB PLAs at 130 °C under the quiescent and shear conditions (the fixed shear rate of 1 s^−1^ and various shear times, *t*_s_) are shown in Fig. 3. Note that the curves for LCB PLA8 at *t*_s_ of 50 and 60 s are not measurable due to the much high normal stress in the pre-shear process. *G*′ evolves from an initial plateau value with the growing crystallites in the supercooled melt and then rapidly rises up to a high plateau value, showing a sigmoidal shape, typical for crystallization of semicrystalline polymers^44^. Note that the final plateau keeps slight raising because the secondary crystallization process continuously undergoes. The induction time of crystallization, *t*_0_ is defined as the intersection between the largest slope of the storage modulus-time curve and the line through the initial plateau of *G*′ (indicated by two green dash lines in Fig. 3a)^21^. It is evidently seen from Fig. 3 that for all the PLA samples the application of shear with the shear rate of 1 s^−1^ and shear time of 5 s leads to a dramatic decrease in *t*_0_ as compared with that at the quiescent condition. A continuous decrease in *t*_0_ can be seen when the shear time increases from 5 to 60 s for linear PLA and LCB PLA2, and eventually a saturation in *t*_0_ for LCB PLAs with higher branching degrees can be seen at the long shear time. The acceleration of the overall crystallization process can be identified by shifting of the storage modulus curves to the lower time side. However, two characteristic accelerating behaviors in the overall crystallization process can be clearly distinguished from Fig. 3: (i) for linear PLA and LCB PLA2, the storage modulus curves shift to the lower time side with the shape of the curves being similar to that at the quiescent condition; (ii) for LCB PLA4, LCB PLA6 and LCB PLA8, as the shear time increases, not only the storage modulus curves shift to the lower time side, but also their shapes change (showing decrease in the slope with increasing shear time). Housmans *et al.*^37^ observed a similar phenomenon in their study on isotactic polypropylene (iPP) and advocated that the shift of storage modulus curves with similar slopes indicated an increase in number of nuclei, while the shift of storage modulus curves with the slope change suggested the decrease in crystal growth dimension, i.e., a transformation from the spherulitic morphology to shish-kebabs growing in two-dimension off the fibrillar nuclei. The definite relationship between the storage modulus curves and the actual morphologies for the present work will be discussed in the later sections. The variations of plateau modulus deserve an attention. It is inevitable that the fluctuations of the ending plateau modulus occur due to the variations of the sample thickness as a result of sample shrinkage during crystallization, as in the case for linear PLA and LCB PLA2. Nevertheless, the storage modulus curves for LCB PLA4, LCB PLA6 and LCB PLA8 exhibit evident decreases in the ending plateau modulus with increasing shear time, which cannot be considered to result from the fluctuations as above discussed. Note that the validity of the above phenomenon was proved by the repeated tests. The substantial drop in the ending plateau modulus is probably attributed to the reduced crystallinity for LCB PLAs^45^ with relatively high branching degrees as the *t*_s_ increases at the shear rate of 1 s^−1^.

**Figure 4:**
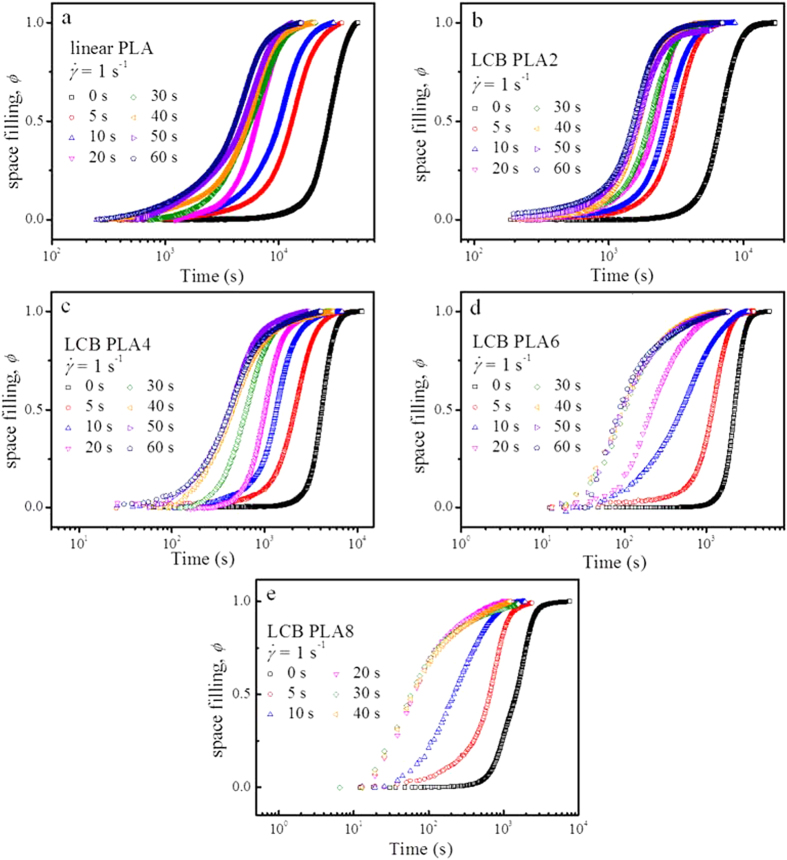
Evolutions of space filling, *ϕ* during isothermal crystallization at 130 °C for (**a**) linear PLA, (**b**) LCB PLA2, (**c**) LCB PLA4, (**d**) LCB PLA6, and (**e**) LCB PLA8, respectively, under the quiescent and various shear conditions.

To quantitatively evaluate the overall crystallization kinetics for linear PLA and LCB PLAs under the quiescent and different shear conditions, the crystallization half-time, *t*_1/2_ was determined. From the rheological perspective, the degree of space filling suggests the transformed fraction of the crystalline phase, which can be adopted to characterize the crystallization kinetics. Here the space filling, *ϕ* is estimated by normalizing the *G*′ data in Fig. 4 according to the method proposed by Pogodina *et al.*^46^.


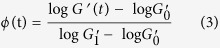


with 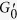 and 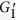 are the starting and ending plateau modulus values, respectively. As abovementioned, the ending plateau modulus is not horizontal. Herein, 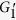 is defined as the intersection of the two tangents of extrapolated end plateau and the regime with the fastest increase in *G*′. The changes of *ϕ* with time at different shear conditions for linear PLA and LCB PLAs are shown in Fig. 4. It can be clearly found that for LCB PLAs with higher branching degree (see Fig. 4c,d,e), the time evolution curves of *ϕ* nearly overlap when long shear time is applied at the constant shear rate of 1 s^−1^, indicating a saturation effect of shear time on crystallization kinetics for LCB PLA.

**Figure 5:**
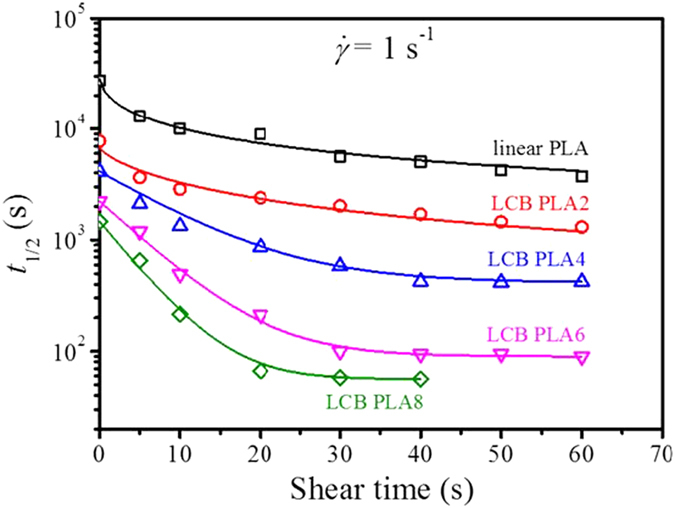
Changes of crystallization half-time, *t*_1/2_ with shear time, *t*_s_ at the shear rate of 1 s^−1^ at 130 °C for linear PLA and LCB PLAs.

To well elucidate the effects of shear time, *t*_s_ on the overall crystallization kinetics for linear PLA and LCB PLAs with different branching degrees, the changes of *t*_1/2_ with *t*_s_ at the shear rate of 1 s^−1^ are displayed in Fig. 5. It is evident that under quiescent condition *t*_1/2_ decreases obviously by more than 1 order of magnitude from linear PLA to LCB PLA8. With the application of shear and increase in *t*_s_ more significant decrease of *t*_1/2_ by more than 2 orders of magnitude occurs, indicating a facilitating effect of shear on acceleration of the crystallization kinetics through increase in the long chain branching degree. Furthermore, the decreases of *t*_1/2_ with increasing *t*_s_ for linear PLA and LCB PLAs behave evidently different. For linear PLA a shear at 1 s^−1^ for *t*_s_ of 5 s leads to a rapid drop in *t*_1/2_ and further increases in *t*_s_ to 10 s or more merely achieve a modest decrease in *t*_1/2_. The changing trend of *t*_1/2_ with *t*_s_ for LCB PLA2 quite resembles that for linear PLA. Nevertheless, the *t*_1/2_ values for LCB PLA4 undergo a continuously rapid decrease and eventually reach a plateau region, indicating a saturation effect of *t*_s_ on acceleration of the crystallization kinetics. LCB PLA6 and LCB PLA8 behave similarly as LCB PLA4. The apparent difference is that for LCB PLA6 and LCB PLA8 *t*_1/2_ decreases much faster and the plateau region appears at much shorter *t*_s_. The saturation occurs at *t*_s_ of 40 s for LCB PLA4, at *t*_s_ of 30 s for LCB PLA6 and at *t*_s_ of 20 s for LCB PLA8, suggesting that the higher the branching degree, the faster the saturation of crystallization kinetics for LCB PLAs. The effect of long chain branching on the enhancement of crystallization kinetic of PLA under the quiescent condition has been reported in the literature and was attributed to the nucleating effect of the branching chains^16^,^18^,^20^. The saturation effect of shear time on acceleration of the crystallization kinetics for LCB PLAs with high branching degrees can be correlated to the joint action of LCB chains and shear flow, which will be discussed in a later section.

### Nucleation densities estimated from rheological measurements

Generally, the polymer crystallization kinetics is primarily dictated by the nucleation and crystal growth processes^47^,^48^. As has been reported in our previous work, the spherulitic growth rates for LCB PLAs under the quiescent crystallization condition are lower than that for linear PLA, which decrease with increasing branching degree at each isothermal crystallization temperature^20^. Therefore, the enhancement of nucleation takes the dominant role for improving the overall crystallization kinetics for LCB PLAs and the nucleation densities are expected to be much higher for LCB PLAs than that for linear PLA at the shear conditions.

**Figure 6:**
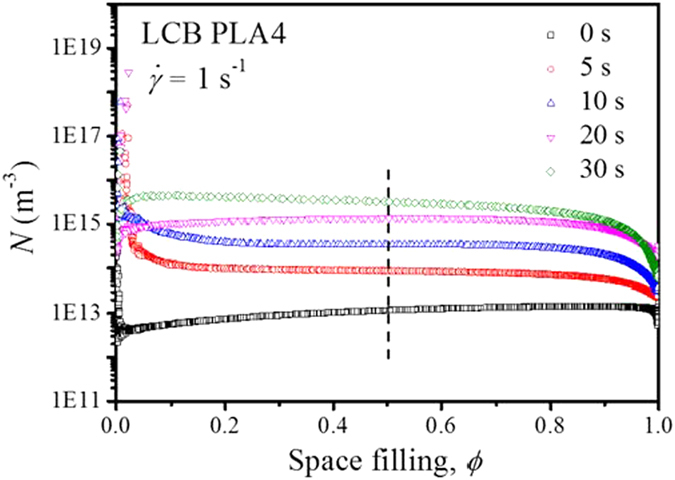
Changes of nucleation density, *N* as functions of space filling, *ϕ* for LCB PLA4 during isothermal crystallization at 130 °C after pre-shear at the shear rate of 1 s^−1^ with different shear times.

By modeling the crystallizing linear PLA and LCB PLAs as a suspension-like systems, the nucleation densities, *N* can be estimated from the space filling, *ϕ*, with an assumption that all nuclei appear after the cessation of pre-shear by using the Avrami equation^49^,^50^:


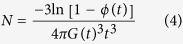


where *G* (*t*) is the spherulitic growth rate. However, this method is restricted to the measurements with relatively short shear time, in which only point-like nucleation takes place. As indicated by the morphological characteristics during the early stage of crystallization as shown in the next section, not only spherulitic morphology but also oriented cylindrical crystalline morphology is induced at the shear time of 40 and 30 s for LCB PLA4 and LCB PLA6, respectively. The spherulitic growth rates for linear PLA and LCB PLAs at the isothermal crystallization temperature of 130 °C have been determined in our previous work^20^ and the values are listed in Table 1. Note that the spherulitic growth rate values are almost unaltered for each PLA sample because in all the measurements the crystal growth process starts after the cessation of shear flow^51^. The changes of nucleation density as functions of space filling for LCB PLA4 as a typical example is shown in Fig. 6. The results for linear PLA and other LCB PLAs can be found in Supplementary Fig. S1. Usually, *N* varies greatly due to the variations of storage modulus at low and high *ϕ* values, while between *ϕ* values of 0.1 and 0.9, *N* keeps almost constant. The *N* value can be determined by either averaging between *ϕ* values of 0.1 and 0.9 or taking the value at the *ϕ* value of 0.5. We choose the values at *ϕ* of 0.5 as *N* for all the PLA samples under the quiescent and various shear conditions in this study.

**Figure 7:**
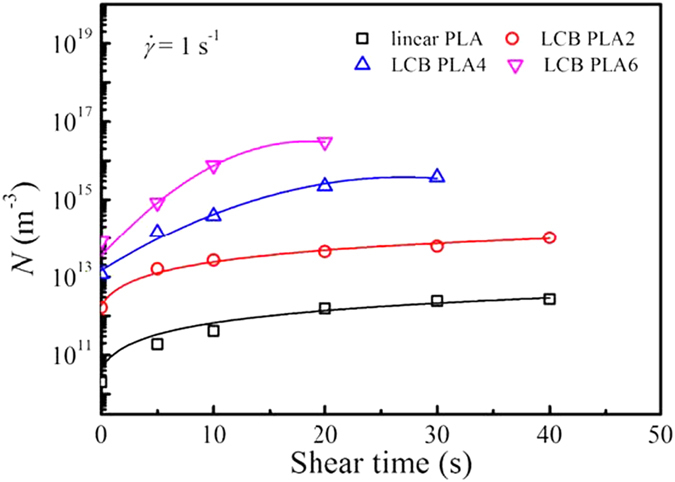
Changes of nucleation density, *N* as functions of shear time, *t*_s_ for linear PLA and LCB PLAs during isothermal crystallization at 130 °C after pre-shear at the shear rate of 1 s^−1^ with different shear times.

The changes of nucleation density, *N* as functions of shear time for linear PLA and LCB PLAs with different branching degrees are shown in Fig. 7. The *N* values for LCB PLA8 are not estimated due to the uncertainty of spherulitic growth rate. In general, the change of *N* with shear time shows an inverse trend as compared with that of the crystallization half-time, *t*_1/2_, i.e., the low value of *t*_1/2_ corresponds to the high value of *N* at the same shear time. For all the PLA samples the application of shear with the short shear time of 5 and 10 s can dramatically improve the *N*. With further increasing of the shear time, the increase in *N* becomes sluggish. Specially, the *N* values for LCB PLA4 and LCB PLA6 show the approach of saturation when the shear time reaches 30 and 20 s, respectively, while the *N* values for linear PLA and LCB PLA2 show gradual increases with shear time without a saturation effect, at least in the shear time window adopted in the present work. Under the quiescent condition the *N* is improved by about 3 orders of magnitude from linear PLA to LCB PLA6, while under the shear conditions with shear time of 20 s, the *N* is improved by almost 5 orders of magnitude. The reliability of the nucleation density derived from the Avrami modeling on the rheological data deserves further verification because equation (4) is sensitive to both space filling and spherulitic growth rate. Therefore, the number of nuclei is counted directly from optical micrographs and then transformed to volume nucleation density, *N*_v_. The detailed nucleation densities derived from these two methods are displayed in Supplementary Fig. S2, suggesting the acceptable reliability of the rheological method in determining the nucleation density. Generally, shear flow strongly enhances the formation of point-like nuclei for linear PLA. The more dominant shear effect on enhancement of nucleation for LCB PLAs suggests the crucial role of LCB in mediating the macromolecular relaxation after the cessation of shear flow, which favors the stabilization for the precursor of nuclei. The higher the branching degree in LCB PLAs, the more stabilized the nuclei. The approach of saturation for nucleation density together with the saturation of crystallization half-time for LCB PLA4 and LCB PLA6, which are directly related to the decrease in slope for the time evolution curves of storage modulus with long shear time, are usually coupled with the decrease in crystal dimension, i.e., from point-like to two-dimensional disk-like or even fibrillar nuclei^37^.

### Crystalline morphologies for linear PLA and LCB PLAs under quiescent and shear conditions

**Figure 8:**
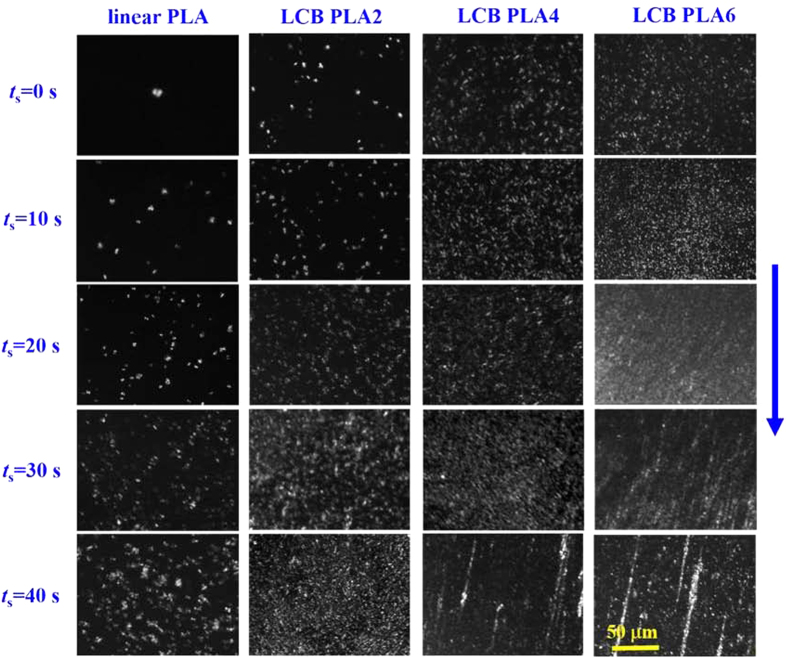
Selected polarized optical micrographs taken at the early stage of crystallization for linear PLA and LCB PLAs at 130 °C after pre-shear with the shear rate of 1 s^−1^ for different shear times. The blue arrow indicates the shear direction.

Polarized optical microscope was used to examine the crystalline morphologies under the quiescent and shear conditions at *T*_c_ of 130 °C for linear PLA and LCB PLAs. The selected polarized optical micrographs taken at the early crystallization stage are shown in Fig. 8. It can be observed that under the quiescent condition the introduction of LCB increases the nucleation density. This result accords well with our previous finding that the LCB structure plays a role of nucleating agent, which lowers the free energy barrier of nucleation for linear PLA precursor^20^. With the application of pre-shear at the shear rate of 1 s^−1^ for the shear time of 10 s, the nucleation densities for all the PLA samples are increased. With further increase in shear time, linear PLA shows a gradual increase of nucleation density. A unique phenomenon can be found for LCB PLAs under the pre-shear conditions with long shear times, for which oriented cylindrical crystals are present and dispersed among the dense point-like nuclei. This result has not been extensively reported in the literature for LCB PLAs. It is further obviously seen that for LCB PLA with high branching degree the shear time required for the formation of oriented crystalline morphology becomes shorter than that for LCB PLA with low branching degree. The critical shear times for the formation of distinct oriented crystalline morphology for LCB PLA4 and LCB PLA6 are 40 and 30 s, respectively, while for LCB PLA2 the distinct oriented crystalline morphology is not observed in the shear time range for the optical microscope observation. The crystalline morphological transformation from the point-like nuclei to oriented crystalline morphology is highly consistent with the description about the rheological results.

**Figure 9:**
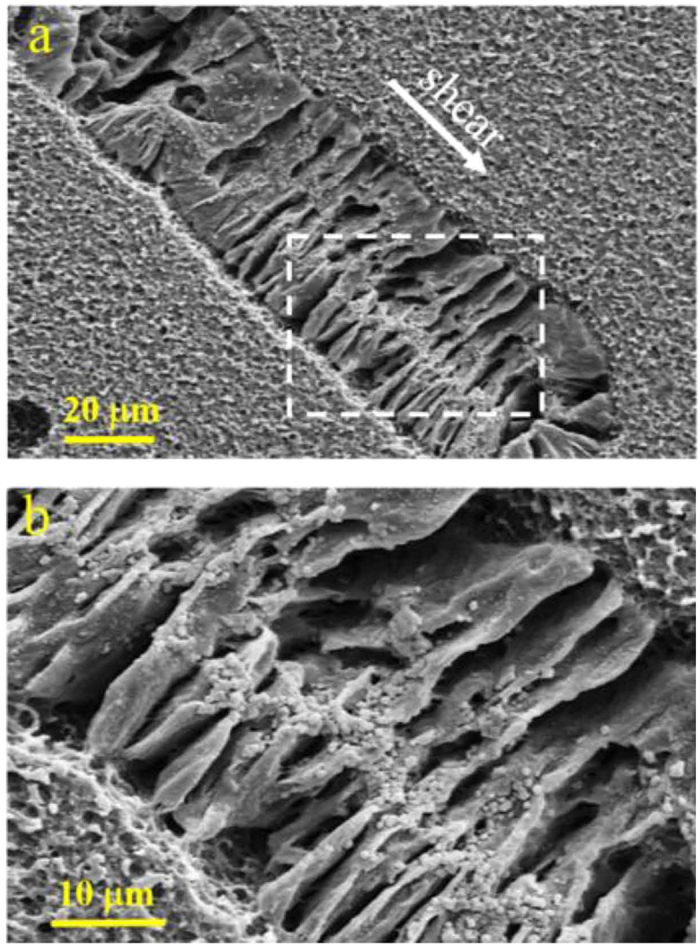
SEM observation of LCB PLA4 after crystallization at 130 °C for 1 h with the shear rate of 1 s^−1^ and the shear time of 40 s. (**a**) An overview of the shear-induced cylindrical crystalline morphology. (**b**) Micrograph in higher magnification for the white rectangular portion of (**a**). The white arrow in (**a**) indicates the shear direction.

The microstructure for the shear-induced oriented crystalline morphology was further examined by using scanning electron microscopy (SEM). Figure 9 shows the crystalline morphology in LCB PLA4 film crystallized at 130 °C for 1 h after sheared at the shear rate of 1 s^−1^ for the shear time of 40 s. It is clearly seen that the oriented cylindrical crystalline morphology resembles a shish-kebab morphology, with oriented lamellae growing perpendicular to the shear direction. However, highly oriented fibrillar shish core is absent in the cylindrical structures, which is attributed to the weak shear intensity at the shear rate of mere 1 s^−1^. The symmetrically developed shish-kebab-like structure is probably induced by the long shear time of 40 s, during which the compact row nuclei aligned along the shear direction. These compact aligned nuclei give rise to the subsequent lamellar growth perpendicular to the shear direction. The above result illustrates the favorable formation of oriented cylindrical crystalline morphology resembling a shish-kebab for PLA under a weak shear condition for a long shear time with the assistance of a LCB structure.

### Mechanism for transformation from spherulitic to oriented crystalline morphologies under shear flow

**Figure 10:**
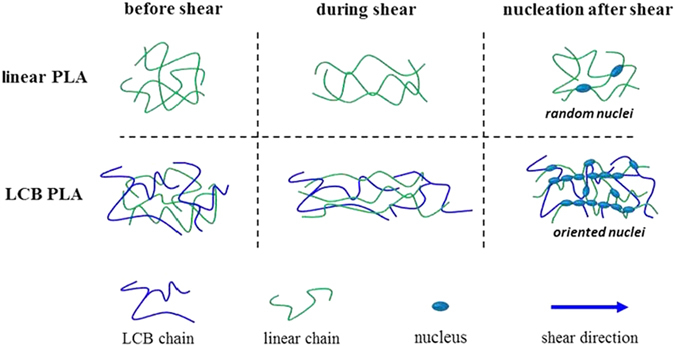
Schematic illustration for the formation of spherulitic and oriented crystalline morphologies for linear PLA and LCB PLAs, respectively.

The shear-induced crystallization behavior of PLA differs greatly from that for polyolefins, such as polypropylene (PP) and polyethylene (PE), because the macromolecular chains of PLA are short and semirigid and suffer from undesired fast relaxation after shear^52^,^53^. Consequently, the shish-kebab structure is not easily formed and has been rarely reported in PLA unless the methods with intense shear flows are adopted, such as the oscillation shear injection molding technique used by Xu *et al.*^52^. In our case the shear rate of 1 s^−1^ is too low to achieve the shish-kebab formation. Instead the oriented cylindrical crystalline morphology is induced by shearing with long durations^54^. The oriented crystalline morphology is actually composed of compact point-like nuclei, which orientate along the shear direction^54^,^55^,^56^. The shear-induced nucleation results from a competition from chain orientation, stretching during shear and relaxation after the cessation of shear. It has been discussed that the relaxation of LCB PLAs is restrained as evidenced by the broadened relaxation spectrum and increased longest relaxation time, *τ*^HMW^ with increasing branching degree. Therefore, shear-induced crystalline morphologies of LCB PLAs evolve quite differently from that of linear PLA. The possible mechanism is schematically provided in Fig. 10. For linear PLA shear flow causes the entangled macromolecular chain network to be stretched. However, only few oriented chain segments survive due to the fast relaxation of the stretched network^21^,^31^,^44^,^52^,^53^. Thus, separated point-like nuclei are induced even if a long shear time of 40 s is used. As for LCB PLAs the entangled macromolecular chain networks are composed not only by linear chain entanglements but also the entanglements from both linear and branched long chains. After the cessation of shear, the much slow relaxation behavior of the LCB chains hinders the relaxation of the stretched linear chain networks. Consequently, large amounts of oriented chain segments, which act as precursors for crystallization, can be stabilized and then transformed to compact nuclei entities. The oriented cylindrical crystalline morphology is thus formed with the densely packed nuclei aligned along the shear direction, which is obviously different from the conventional shish-kebab morphology. For LCB PLAs with high branching degrees the formation of oriented crystalline morphology becomes easier due to much slower relaxation of the stretched chain networks, which can be proved by the polarized optical micrographs shown in Fig. 8. The application of intense shear flow to induce shish-kebab is challenging and intriguing due to both scientific and industrial values, which is now under progress in our group. Although this work concentrates on the shear-induced crystallization of linear PLA and LCB PLAs with different branching degrees with relatively weak shear intensity, the enhancement of crystallization kinetics and transformation of crystalline morphologies concluded mainly from rheological results and optical micrographs may provide potential guidance for industrial processing for PLA, especially LCB PLAs to achieve products with excellent performances.

## Conclusions

A series of long chain branched polylactides (LCB PLAs) with different long chain branching degrees was prepared by *γ* irradiation after melt blending of linear PLA with trimethylolpropane triacrylate (TMPTA) of different amounts. The dynamic frequency sweep tests indicate the broadened melt relaxation spectra for LCB PLAs, especially in the longest relaxation time region. By tracking the storage modulus evolutions the crystallization kinetics for these LCB PLAs can be evaluated. The crystallization half-time is obtained by modeling the crystallizing LCB PLAs as a suspension-like system. A saturation in shear time is observed for the crystallization kinetics of LCB PLAs and the shear time at saturation decreases with increasing branching degree. The nucleation density is obtained by using the Avrami equation from space filling, with the saturation effect in shear time on the enhancement of nucleation density presented as well. The saturation effect well corresponds to the transformation of spherulitic to oriented crystalline morphologies as observed from the polarized optical micrographs for LCB PLAs with sufficient shear time. The formation mechanism of the oriented crystalline morphology for LCB PLAs under shear flow is related to the hindering of relaxation of the stretched macromolecular chain network for LCB chains. The significant enhancement in crystallization kinetics and morphological transformation for LCB PLAs with different branching degrees lead to improved production efficiency, providing a potential guidance for manufacturing PLA products with preferable mechanical properties.

## Experimental Section

### Sample preparation

A commercial available polylactide (PLA) (model 2002D, Natureworks) with 96 wt% of L-isomeric content was used in this work. The long chain branched PLA (LCB PLA) samples were prepared through *γ* irradiation. The detailed preparation procedure can be found elsewhere^15^,^20^,^21^. Briefly, a trifunctional monomer, trimethylolpropane triacrylate (TMPTA) of different amounts was melt blended with linear PLA, and then the blends were subjected to *γ* irradiation of 5 kGy (2.2 kGy/h). The sample codes, LCB PLA2, LCB PLA4, LCB PLA6 and LCB PLA8 refer to the irradiated PLA with TMPTA amounts of 0.2, 0.4, 0.6 and 0.8 wt%, respectively. Linear PLA was subjected to the same thermal treatment but with no *γ* irradiation. The preparation formula and molecular mass parameters obtained in our previous work^14^,^20^ are listed in Table 2. The branching degrees were characterized by the LCB contents.

### Measurements of dynamic viscoelastic properties

Dynamic viscoelastic properties were determined from small amplitude oscillatory shear measurements using parallel plate rotational rheometer (TA-AR2000EX, TA Instruments) with geometry of 25 mm in diameter and gap of 1 mm at the constant temperature of 180 °C. The measurements were carried out in the linear viscoelastic regime from frequency of 500 to 0.1 rad/s for linear PLA and 500 to 0.05 rad/s for LCB PLAs, respectively, with a strain of 2%. All measurements were performed in a nitrogen atmosphere to avoid degradation of the samples.

### Measurements on shear-induced crystallization kinetics

The shear-induced crystallization kinetics was followed using rheometer (TA-AR2000EX, TA Instruments). To avoid transducer instability, parallel plates with 8 mm in diameter were used. The experimental procedure is described as follows. Firstly, linear PLA and LCB PLA samples were kept at 180 °C for 5 min to erase thermal histories. Subsequently, the samples were cooled at 15 °C/min to the desired isothermal crystallization temperature of 130 °C. The pre-shear steps with different shear conditions were performed immediately when 130 °C was reached. The following isothermal crystallization process was monitored through oscillatory tests with an angular frequency of 5 rad/s and a strain of 0.5%, which was low enough to avoid the modification of crystallization kinetics. The thermal and shear protocol can be found in our previous work (Fig. 1 in ref. 21). Note that during the whole test, the gap was automatically adjusted to compensate thermal expansion (shrinkage) of the samples.

### Morphological evolutions observed by POM

A polarized optical microscopy (POM, Olympus BX51, Japan) equipped with a Linkam CSS-450 hot stage (Linkam Scientific Instruments, UK) was used for observation of the morphological evolutions during crystallization under quiescent and shear conditions for linear PLA and LCB PLA samples. Before each test, the two quartz plates were carefully cleaned. A small piece of the film sample was loaded to the shear cell and heated to 200 °C and then the gap was immediately lowered to 50 μm. The sample was kept for 5 min to erase thermal history, and then cooled to 130 °C at 15 °C/min. Shear was applied once the temperature reached 130 °C. After the cessation of shear, optical micrographs were taken to track the morphological evolutions during quiescent and shear-induced isothermal crystallization process at appropriate time interval until the impingements of crystals. Zero time for the collected micrographs was assigned when shear was just stopped. Most of the measurements were conducted in duplicate, showing sufficient reproducibility for the results.

### Scanning Electron Microscopy (SEM) Observation

For observation of the crystalline morphology, the sheared and crystallized films were etched in a water/methanol (1/2 by volume) solution containing 0.025 mol/L of sodium hydroxide at 25 °C for 24 h. The etched films were subsequently cleaned by distilled water. A field-emission SEM (Sirion200, FEI, USA), operating at the accelerated voltage of 10 kV was used to observe the crystalline morphology of sheared sample, which were sputter coated with gold before observation.

## Additional Information

**How to cite this article**: Wang, J. *et al.* Shear-induced enhancements of crystallization kinetics and morphological transformation for long chain branched polylactides with different branching degrees. *Sci. Rep.* **6**, 26560; doi: 10.1038/srep26560 (2016).

## Supplementary Material

Supplementary Information

## Figures and Tables

**Table 1:** Spherulitic growth rates for linear PLA and LCB PLAs at the isothermal crystallization temperature of 130 °C.

Sample code	Linear PLA	LCB PLA2	LCB PLA4	LCB PLA6	LCB PLA8
*G* (10^−9^ m/s)	7.4	7.0	5.8	5.5	–

The spherulitic growth rate for LCB PLA8 was difficult to obtain from the optical micrographs.

**Table 2:** Preparation formula and molecular mass parameters for linear PLA and LCB PLAs.

Sample code	TMPTA content (wt%)	γ radiation dose (kGy)	*M*_w_ (kg/mol)	*M*_z_ (kg/mol)	*M*_w_/*M*_n_	LCB content (%)
linear PLA	0	0	112	167	1.4	0
LCB PLA2	0.2	5	114	188	1.7	1.2
LCB PLA4	0.4	5	169	299	2.2	6.4
LCB PLA6	0.6	5	269	723	3.3	11.8
LCB PLA8	0.8	5	–	–	–	–

The *M*_w_, *M*_z_ and *M*_w_/M_n_ are determined by SEC-MALLS. Note that the values for LCB PLA8 are outside the measurement limitation. LCB content was obtained by fitting the curves of molar mass distribution^15^.

## References

[b1] Garlotta D. A literature review of poly (lactic acid). J. Polym. Environ. 9, 63–84 (2001).

[b2] Saeidlou S., Huneault M. A., Li H. & Park C. B. Poly (lactic acid) crystallization. Prog. Polym. Sci. 37, 1657–1677 (2012).

[b3] Lim L. T., Auras R. & Rubino M. Processing technologies for poly (lactic acid). Prog. Polym. Sci. 33, 820–852 (2008).

[b4] Sarasua J. R., Prud’Homme R. E., Wisniewski M., Le Borgne A. & Spassky N. Crystallization and melting behavior of polylactides. Macromolecules 31, 3895–3905 (1998).

[b5] Mitomo H., Kaneda A., Quynh T. M., Nagasawa N. & Yoshii F. Improvement of heat stability of poly (L-lactic acid) by radiation-induced crosslinking. Polymer 46, 4695–4703 (2005).

[b6] Li H. & Huneault M. A. Effect of nucleation and plasticization on the crystallization of poly (lactic acid). Polymer 48, 6855–6866 (2007).

[b7] Ghosh S., Viana J., Reis R. & Mano J. Effect of processing conditions on morphology and mechanical properties of injection-molded poly (L-lactic acid). Polym. Eng. Sci. 47, 1141–1147 (2007).

[b8] Tsuji H. & Ikada Y. Properties and morphologies of poly (L-lactide): 1. annealing condition effects on properties and morphologies of poly (L-lactide). Polymer 36, 2709–2716 (1995).

[b9] Krikorian V. & Pochan D. J. Unusual crystallization behavior of organoclay reinforced poly (L-lactic acid) nanocomposites. Macromolecules 37, 6480–6491 (2004).

[b10] Barrau S. *et al.* Crystallization behavior of carbon nanotube-polylactide nanocomposites. Macromolecules 44, 6496–6502 (2011).

[b11] Xu Z. H. *et al.* Enhanced nucleation rate of polylactide in composites assisted by surface acid oxidized carbon nanotubes of different aspect ratios. ACS Appl. Mater. Interfaces 3, 3744–3753 (2011). 21859160 10.1021/am200932q

[b12] Xu Z. H. *et al.* Morphology, rheology and crystallization behavior of polylactide composites prepared through addition of five-armed star polylactide grafted multiwalled carbon nanotubes. Polymer 51, 730–737 (2010).

[b13] Wu D. F., Cheng Y. X., Feng S. H., Yao Z. & Zhang M. Crystallization behavior of polylactide/graphene composites. Ind. Eng. Chem. Res. 52, 6731–6739 (2013).

[b14] Xu H. J., Fang H. G., Bai J., Zhang Y. Q. & Wang Z. G. Preparation and characterization of high-melt-strength polylactide with long-chain branched structure through γ-radiation-induced chemical reactions. Ind. Eng. Chem. Res. 53, 1150–1159 (2014).

[b15] Fang H. G., Zhang Y. Q., Bai J., Wang Z. K. & Wang Z. G. Bimodal architecture and rheological and foaming properties for gamma-irradiated long-chain branched polylactides. RSC Adv. 3, 8783–8795 (2013).

[b16] Wang L. Y. *et al.* Rheology and crystallization of long-chain branched poly (L-lactide)s with controlled branch length. Ind. Eng. Chem. Res. 51, 10731–10741 (2012).

[b17] Wang Y. B. *et al.* Rheological and topological characterizations of electron beam irradiation prepared long-chain branched polylactic acid. J. Appl. Polym. Sci. 122, 1857–1865 (2011).

[b18] Nofar M., Zhu W. L., Park C. B. & Randall J. Crystallization kinetics of linear and long-chain-branched polylactide. Ind. Eng. Chem. Res. 50, 13789–13798 (2011).

[b19] Wang L. Y. *et al.* Blends of linear and long-chain branched poly (l-lactide) s with high melt strength and fast crystallization rate. Ind. Eng. Chem. Res. 51, 10088–10099 (2012).

[b20] Bai J., Fang H. G., Zhang Y. Q. & Wang Z. G. Studies on crystallization kinetics of bimodal long chain branched polylactides. CrystEngComm 16, 2452–2461 (2014).

[b21] Fang H. G., Zhang Y. Q., Bai J. & Wang Z. G. Shear-induced nucleation and morphological evolution for bimodal long chain branched polylactide. Macromolecules 46, 6555–6565 (2013).

[b22] Wang J. Y. *et al.* More dominant shear flow effect assisted by added carbon nanotubes on crystallization kinetics of isotactic polypropylene in nanocomposites. ACS Appl. Mater. Interfaces 7, 1364–1375 (2015). 25569561 10.1021/am507938s

[b23] Agarwal P. K. *et al.* Shear-induced crystallization in novel long chain branched polypropylenes by *in situ* rheo-SAXS and -WAXD. Macromolecules 36, 5226–5235 (2003).

[b24] Somani R. H., Yang L., Zhu L. & Hsiao B. S. Flow-induced shish-kebab precursor structures in entangled polymer melts. Polymer 46, 8587–8623 (2005).

[b25] Heeley E. L. *et al.* Shear-induced crystallization in blends of model linear and long-chain branched hydrogenated polybutadienes. Macromolecules 39, 5058–5071 (2006).

[b26] Kimata S. *et al.* Molecular basis of the shish-kebab morphology in polymer crystallization. Science 316, 1014–1017 (2007). 17510361 10.1126/science.1140132

[b27] Okura M., Mykhaylyk O. O. & Ryan A. J. Effect of matrix polymer on flow-induced nucleation in polymer blends. Phys. Rev. Lett. 110, 087801 (2013). 23473204 10.1103/PhysRevLett.110.087801

[b28] Auhl D. *et al.* Long-chain branched polypropylenes by electron beam irradiation and their rheological properties. Macromolecules 37, 9465–9472 (2004).

[b29] Yu F. Y. *et al.* Flow induced crystallization of long chain branched polypropylenes under weak shear flow. Eur. Polym. J. 45, 2110–2118 (2009).

[b30] García-Franco C. A., Srinivas S., Lohse D. J. & Brant P. Similarities between gelation and long chain branching viscoelastic behavior. Macromolecules 34, 3115–3117 (2001).

[b31] Zhong Y. *et al.* Rheologically determined critical shear rates for shear-induced nucleation rate enhancements of poly (lactic acid). ACS Sustainable Chem. Eng. 1, 663–672 (2013).

[b32] Mykhaylyk O. O. *et al.* The specific work of flow as a criterion for orientation in polymer crystallization. Macromolecules 41, 1901–1904 (2008).

[b33] Nogales A. *et al.* Shear-induced crystallization of isotactic polypropylene with different molecular weight distributions: *in situ* small- and wide-angle X-ray scattering studies. Polymer 42, 5247–5256 (2001).

[b34] Duplay C., Monasse B., Haudin J. M. & Costa J. L. Shear-induced crystallization of polypropylene: influence of molecular weight. J. Mater. Sci. 35, 6093–6103 (2000).

[b35] Ogino Y. *et al.* Effects of high molecular weight component on crystallization of polyethylene under shear flow. Polymer 47, 5669–5677 (2006).

[b36] Seki M., Thurman D. W., Oberhauser J. P. & Kornfield J. A. Shear-mediated crystallization of isotactic polypropylene: the role of long chain-long chain overlap. Macromolecules 35, 2583–2594 (2002).

[b37] Housmans J. W., Steenbakkers R. J. A., Roozemond P. C., Peters G. W. M. & Meijer H. E. H. Saturation of pointlike nuclei and the transition to oriented structures in flow-induced crystallization of isotactic polypropylene. Macromolecules 42, 5728–5740 (2009).

[b38] van Meerveld J., Peters G. W. M. & Hütter M. Towards a rheological classification of flow induced crystallization experiments of polymer melts. Rheol. Acta 44, 119–134 (2004).

[b39] Yan T. *et al.* Critical strain for shish-kebab formation. Macromolecules 43, 602–605 (2009).

[b40] Khanna Y. P. Rheological mechanism and overview of nucleated crystallization kinetics. macromolecules 26, 3639–3643 (1993).

[b41] Boutahar K., Carrot C. & Guillet J. Crystallization of polyolefins from rheological measurements relation between the transformed fraction and the dynamic moduli. Macromolecules 31, 1921–1929 (1998).

[b42] Ma Z., Steenbakkers R. J. A., Giboz J. & Peters G. W. M. Using rheometry to determine nucleation density in a colored system containing a nucleating agent. Rheol. Acta 50, 909–915 (2011).

[b43] Meerveld J., Peters G. W. M. & Hütter M. Towards a rheological classification of flow induced crystallization experiments of polymer melts. Rheol. Acta 44, 119–134 (2004).

[b44] Wang Z. G., Hsiao B. S., Sirota E. B., Agarwal P. & Srinivas S. Probing the early stages of melt crystallization in polypropylene by simultaneous small- and wide-angle X-ray scattering and laser light scattering. Macromolecules 33, 978–989 (2000).

[b45] Dorgan J. R., Lehermeier H. & Mang M. Thermal and rheological properties of commercial-grade poly (lactic acid)s. J. Polym. Environ. 8, 1–9 (2000).

[b46] Pogodina N. V., Winter H. H. & Srinivas S. Strain effects on physical gelation of crystallizing isotactic polypropylene. J. Polym. Sci., Part B: Polym. Phys. 37, 3512–3519 (1999).

[b47] Janeschitz-Kriegl H., Ratajski E. & Stadlbauer M. Flow as an effective promotor of nucleation in polymer melts: a quantitative evaluation. Rheol. Acta 42, 355–364 (2003).

[b48] Janeschitz-Kriegl H. & Eder G. Shear induced crystallization, a relaxation phenomenon in polymer melts: a re-collection. J. Macromol. Sci., Part B: Phys. 46, 591–601 (2007).

[b49] Avrami M. Kinetics of phase change. I. General theory. J. Chem. Phys. 7, 1103–1112 (1939).

[b50] Avrami M. Kinetics of phase change. II. Transformation-time relations for random distribution of nuclei. J. Chem. Phys. 8, 212–224 (1940).

[b51] Koscher E. & Fulchiron R. Influence of shear on polypropylene crystallization: morphology development and kinetics. Polymer 43, 6931–6942 (2002).

[b52] Xu H. *et al.* Formation of shish-kebabs in injection-molded poly (l-lactic acid) by application of an intense flow field. ACS Appl. Mater. Interfaces 4, 6774–6784 (2012). 23153180 10.1021/am3019756

[b53] Tang H. *et al.* Shear flow and carbon nanotubes synergistically induced nonisothermal crystallization of poly (lactic acid) and its application in injection molding. Biomacromolecules 13, 3858–3867 (2012). 23072455 10.1021/bm3013617

[b54] Li X. J., Li Z. M., Zhong G. J. & Li L. B. Steady-shear-induced isothermal crystallization of poly (L-lactide) (PLLA). J. Macromol. Sci., Part B: Phys. 47, 511–522 (2008).

[b55] Zhang C. G. *et al.* Formation of cylindrite structures in shear-induced crystallization of isotactic polypropylene at low shear rate. Polymer 48, 1105–1115 (2007).

[b56] Huang S. Y., Li H. F., Jiang S. C., Chen X. S. & An L. J. Crystal structure and morphology influenced by shear effect of poly (L-lactide) and its melting behavior revealed by WAXD, DSC and *in-situ* POM. Polymer 52, 3478–3487 (2011).

