# Supplementary material for: Shear-induced enhancements of crystallization kinetics and morphological transformation for long chain branched polylactides with different branching degrees
Source: Sci Rep. 2016 Jun 1;6:26560. doi: 10.1038/srep26560 (PMC4887888; doi:10.1038/srep26560)
Supplement: Supplementary Information [file srep26560-s1.pdf]

Supplementary Information

Shear-induced enhancements of crystallization kinetics and morphological transformation for long chain branched polylactides with different branching degrees

Junyang Wang,[1] Jing Bai,[1] Yaqiong Zhang,[1] Huagao Fang,[2] & Zhigang Wang[1,*]

[1]CAS Key Laboratory of Soft Matter Chemistry, Department of Polymer Science and Engineering, Hefei National Laboratory for Physical Sciences at the Microscale, University of Science and Technology of China, Hefei, Anhui Province 230026, P. R. China

[2]Provincial Key Laboratory of Advanced Functional Materials and Devices, Institute of Polymer Materials and Chemical Engineering, School of Chemistry and Chemical Engineering, Hefei University of Technology, Hefei, Anhui Province 230009, P. R. China

* zgwang2@ustc.edu.cn

Determination of the critical shear rate for linear PLA at the crystallization temperature of 130 °C. The longest reptation time, τ_{rep}^{HMW} for linear PLA at 180 °C has been obtained from the relaxation time spectrum shown in Figure 3 in the main manuscript. The Rouse time (stretch relaxation time), τ_R can be calculated using the relation based on the Tube model of Doi and Edwards:[1]

$$\tau_R = \frac{\tau_{rep}}{3Z} \qquad \text{(S1)}$$

where Z is the average number of entanglements (Z = M_w/M_e, M_e is the molecular mass

between entanglements). The calculated Z value for linear PLA in the present work is about 11 by using the M_e value of 8000 g/mol for PLA. Table S1 lists the longest relaxation times together with the characteristic shear rates for the transitions between the orientation and the stretch regimes for linear PLA at 180 °C and 130 °C, respectively. The relaxation times and corresponding characteristic shear rates at 130 °C were calculated using the Arrhenius type of temperature dependence (with E_a = 77 kJ/mol[2]), from which the shear conditions for the present study on crystallization kinetics of linear PLA can be chosen.[3,4] The shear rate of 1 s^{-1} was used in the present work, which is not sufficient for the molecular chain stretching of linear PLA at 130 °C. However, due to the much higher longest reptation time of LCB PLAs as compared with linear PLA, the critical shear rate for the chain stretching can be far lower than 1.1 s^{-1}. The chosen shear rate of 1 s^{-1} is sufficient to guarantee that the Weissenberg number, Wi_s > 1.

T (°C)	τ_{rep}^{HMW} (s)	τ_R^{HMW} (s)	$\dot{\gamma}_{I\to II}$ (s^{-1})	$\dot{\gamma}_{II\to III}$ (s^{-1})
180	2.39	0.072	0.41	13.89
130	30.13	0.91	0.03	1.1

Supplementary Table S1. Longest reptation time and rouse time determined from relaxation spectrum and corresponding critical shear rates for the orientation stretch regime transition. $\dot{\gamma}_{I\to II}$ and $\dot{\gamma}_{II\to III}$ are the critical values of shear rate for the transition of flow regimes for linear PLA between Regime I and II, and Regime II and III, respectively.

Changes of nucleation density with space filling obtained from the Avrami equation.

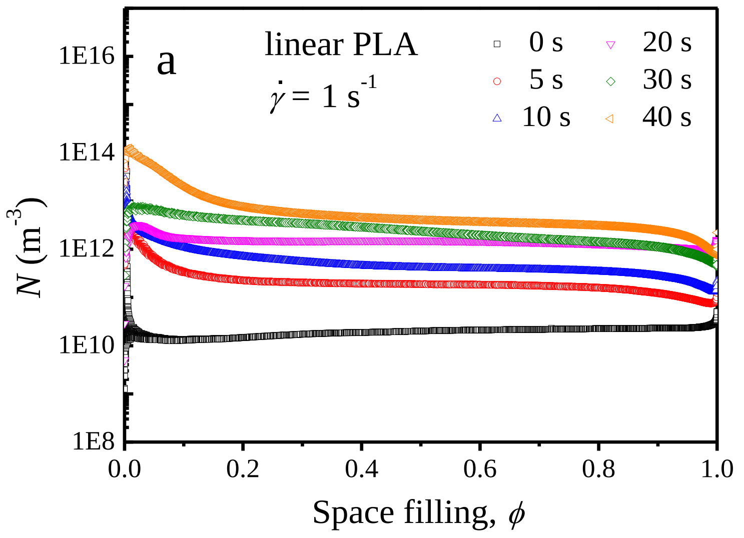

a
linear PLA
$\dot{\gamma}$ = 1 s^{-1}
0 s
5 s
10 s
20 s
30 s
40 s
1E16
1E14
1E12
1E10
1E8
N (m^{-3})
0.0
0.2
0.4
0.6
0.8
1.0
Space filling, ϕ

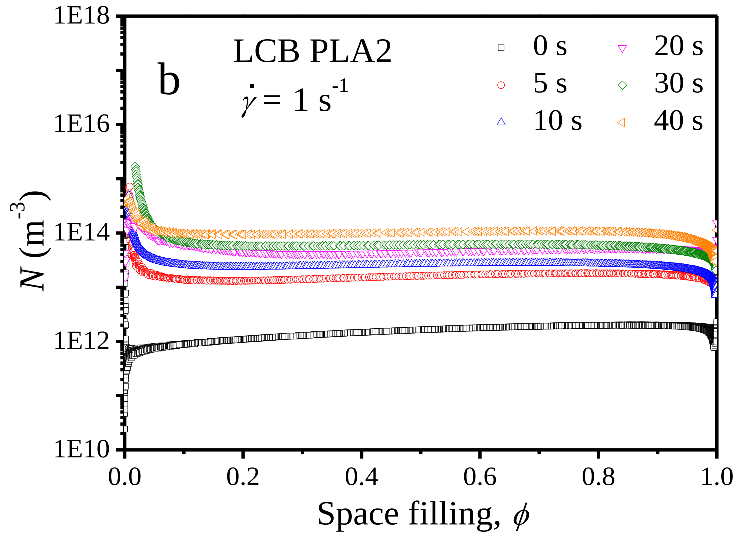

b
LCB PLA2
$\dot{\gamma}$ = 1 s^{-1}
0 s
5 s
10 s
20 s
30 s
40 s
1E18
1E16
1E14
1E12
1E10
N (m^{-3})
0.0
0.2
0.4
0.6
0.8
1.0
Space filling, ϕ

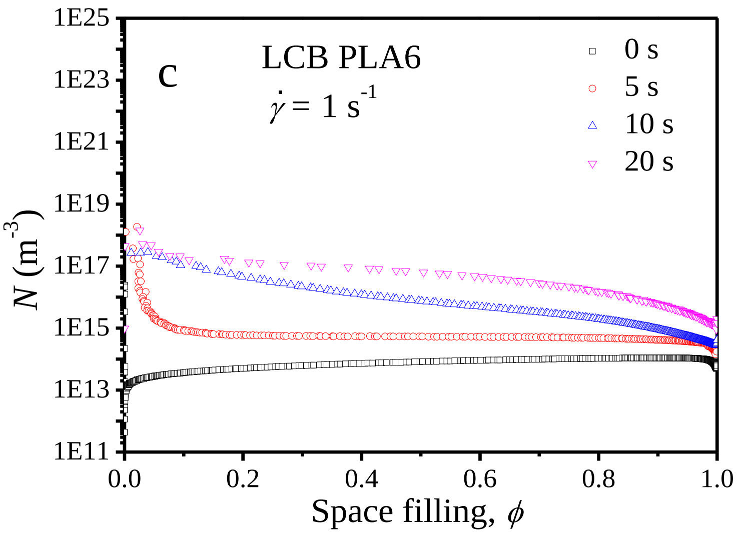

Supplementary Figure S1. Changes of nucleation density, N as functions of space filling, ϕ for (**a**) linear PLA, (**b**) LCB PLA2, and (**c**) LCB PLA6 during crystallization at 130 °C after pre-shear at different shear times with the shear rate of 1 s^{-1}.

Comparison between nucleation densities derived from rheometry and optical microscopy. The determination of nucleation density from the Avrami equation using rheometry is sensitive to space filling obtained from time evolution of storage modulus and spherulitic growth rate. To evaluate the reliability of this method, we counted the number of nuclei, the area nucleation density N_A, from optical micrographs at the early stage of crystallization under the same shear condition as in the rheological measurements. The N_A values were then transformed to volume nucleation density, N_v values using a simple approximation according to equation (S2):

$$N_v = \frac{N_A}{AH} \qquad \text{(S2)}$$

where A is the observed sample area and H is the sample thickness (50 μm in the present work). The N_v values as obtained directly form optical micrographs can be compared to the N values quantified by rheometry. The result is displayed in Fig. S2. It is found that the N values determined from rheometry are in good accordance with that obtained from POM, suggesting the reliability of the quantification of N from rheometry.

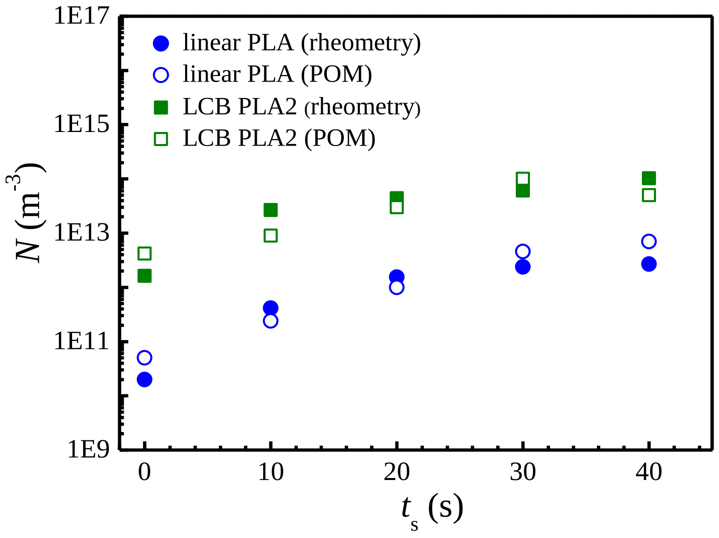

Supplementary Figure S2. Changes of volume nucleation density, *N* as functions of shear time for linear PLA and LCB PLA2 during crystallization at 130 °C after pre-shear with shear rate of 1 s^{-1}. The nucleation densities determined from both rheometry and POM are displayed and compared.

References

1. Macosko, C. W. & Larson, R. G. Rheology: principles, measurements, and applications. (1994).

2. Fang, H. G., Zhang, Y. Q., Bai, J., Wang, Z. K. & Wang, Z. G. Bimodal architecture and rheological and foaming properties for gamma-irradiated long-chain branched polylactides. *RSC Adv.* **3**, 8783-8795 (2013).

3. Zhong, Y. *et al.* Rheologically determined critical shear rates for shear-induced nucleation rate enhancements of poly (lactic acid). *ACS Sustainable Chem. Eng.* **1**, 663-672 (2013).

4. Fang, H. G., Zhang, Y. Q., Bai, J. & Wang, Z. G. Shear-induced nucleation and morphological evolution for bimodal long chain branched polylactide. *Macromolecules* **46**, 6555-6565 (2013).
